# Navigating Information Technologies in Everyday Psychiatry Practice: A Guide for Early Career Psychiatrists

**DOI:** 10.1192/j.eurpsy.2024.131

**Published:** 2024-08-27

**Authors:** K. Vasilchenko

**Affiliations:** Human Artificial Control Keren (HACK) lab, Bar-Ilan University, Safed, Israel

## Abstract

**Abstract:**

This presentation provides an overview of the impact of information technologies on contemporary psychiatric practice, focusing on resources and strategies beneficial for early career psychiatrists. Considering the increasing role of digital technologies in diagnosing and treating mental disorders, the presentation emphasizes the practical applications of artificial intelligence (AI) and machine learning. These technologies offer novel approaches for analyzing large volumes of clinical data, enhancing diagnostic accuracy, and personalizing treatment.

The presentation further examines ethical and legal issues associated with using digital technologies in psychiatry, including ensuring data confidentiality and complying with patient rights. The importance of developing competencies in information security and ethical principles when using digital tools is highlighted.

The talk concludes with an overview of the current and future trends in the use of digital technologies in psychiatry, including the development of virtual therapeutic environments and mobile applications for monitoring and supporting mental health. Examples of successful integration of these technologies into clinical practice are presented, emphasizing their potential to improve the quality of patient care.

Overall, the presentation underscores the importance for early career psychiatrists of mastering information technologies, highlighting their role in enhancing diagnosis, treatment, and patient care, as well as discussing the challenges and opportunities they present.

**Disclosure of Interest:**

None Declared

